# Supplementary material for: Identification of biomarkers associated with clinical severity of chronic obstructive pulmonary disease
Source: PeerJ. 2020 Dec 10;8:e10513. doi: 10.7717/peerj.10513 (PMC7733647; doi:10.7717/peerj.10513)
Supplement: Supplemental Information 2 [file peerj-08-10513-s002.docx]

**Supplementary Tables**

| **Supplementary table 1 Clinical characteristics of all the subjects in GSE database** | | | | |
| --- | --- | --- | --- | --- |
| **Parameters** | **Stage I (n=90)** | **Stage II (n=58)** | **Stage III (n=55)** | **Stage IV (n=13)** |
| Age (years) | 63.29 ± 3.84 | 64.16 ±5.56 | 63.31 ± 7.08 | 65.14 ± 6.22 |
| Gender (Male %) | 61.11 | 65.51 | 69.09 | 69.23 |

| **Supplementary table 2 Clinical characteristics of all the subjects in our own cohort** | | | | |
| --- | --- | --- | --- | --- |
| **Parameters** | **Non-smoker (n=33)** | **Smokers (n=42)** | **GOLD I-II (n=41)** | **GOLD III-IV (n=36)** |
| Age (years) | 60.15 ± 4.95 | 58.44 ±11.23 | 61.52 ± 9.38 | 63.30 ± 7.03 |
| Gender (Male %) | 45.45 | 50.00 | 46.34 | 58.33 |
| BMI (kg/m^2^) | 22.92 ± 3.46 | 23.42 ± 4.17 | 23.82 ±6.01 | 24.02 ±4.16 |
| Diabetes mellitus (%) | 24.24 | 33.33 | 26.59 | 38.89 |
| Hypertension (%) | 33.33 | 35.71 | 36.59 | 36.11 |
| Smoking status (%) | 54.55 | 83.33 | 68.29 | 77.77 |
| Alcohol consumption (%) | 15.15 | 35.71 | 21.95 | 22.22 |
